# A Deep Learning Approach for Estimating Traffic Density Using Data Obtained from Connected and Autonomous Probes

**DOI:** 10.3390/s20174824

**Published:** 2020-08-26

**Authors:** Daisik Nam, Riju Lavanya, R. Jayakrishnan, Inchul Yang, Woo Hoon Jeon

**Affiliations:** 1 Department of Civil and Environmental Engineering, Institute of Transportation Studies, University of California, Irvine, CA 92697, USA; daisikn@uci.edu (D.N.); rlavanya@uci.edu (R.L.); rjayakri@uci.edu (R.J.); 2 Korea Institute of Civil Engineering and Building Technology, Goyang-si 10223, Korea; cwhoon@kict.re.kr

**Keywords:** traffic density estimation, connected and autonomous probes, radar sensors, deep neural network, long-short term neural network

## Abstract

The focus of this research is on the estimation of traffic density from data obtained from Connected and Autonomous Probes (CAPs). CAPs pose an advantage over expensive and invasive infrastructure such as loop detectors. CAPs maneuver their driving trajectories, sensing the presence of adjacent vehicles and distances to them by means of several electronic sensors, whose data can be used for more sophisticated traffic density estimation techniques. Traffic density has a highly nonlinear nature during on-congestion and queue-clearing conditions. Closed-mathematical forms of the traditional density estimation techniques are incapable of dealing with complex nonlinearities, which opens the door for data-driven approaches such as machine learning techniques. Deep learning algorithms excel in data-rich contexts, which recognize nonlinear and highly situation-dependent patterns. Our research is based on an LSTM (Long short-term memory) neural network for the nonlinearity associated with time dynamics of traffic flow. The proposed method is designed to learn the input-output relation of Edie’s definition. At the same time, the method recognizes a temporally nonlinear pattern of traffic. We evaluate our algorithm by using a microscopic simulation program (PARAMICS) and demonstrate that our model accurately estimates traffic density in Free-flow, Transition, and Congested conditions.

## 1. Introduction

Estimating traffic density is of critical importance in understanding current traffic conditions. Accurate estimation of traffic condition is also important for road congestion mitigation technologies such as ramp metering, variable message signs, and signal control, since congested traffic takes time to recover, so it is necessary to manage traffic before the onset of congestion. Traffic density plays a pivotal role in predicting the onset of traffic congestion. Developing vehicle sensing technologies and the concept of “Internet of things” give many opportunities to measure traffic density more accurately. Traffic density estimation using sensor-equipped probes is emerging as a valuable tool in research and practice [1,2,3,4,5,6]. This is apparent when we consider the fact that modern cars are now being equipped with advanced on-vehicle sensors. These sensors can be camera-vision, lidar and radar, which were originally installed for Advanced Driver Assistance Systems (ADAS). The number of vehicles equipped with these advanced functionalities will significantly increase in the near future, specifically with the emergence of Connected and Autonomous Vehicles (CAVs). A side benefit of these technological advancements is that we can now have a sufficient number of vehicles traveling on the road at any given time to obtain a large amount of sensor data, which can then be harnessed to get much-improved estimates of traffic densities.

The basic functionality of CAPs (Connected and Autonomous Probes) involves various sensing equipment (GPS, radars, and cameras) and computing units that collect and process trajectories of detected vehicles. The following data sets can be obtained from CAPs: (1) the number of vehicles on their sensing area, (2) distance to the leading vehicle, and (3) vehicle miles/hours traveled of detected vehicles. Using only the first data set, one can postulate a naïve approach that linearly approximates the number of sensed vehicles to traffic density. This approach leads to large estimation errors because the unit of the output density (vehicles per square meter) is not easily translated to traffic density, which is measured in vehicles per meter and is a more appropriate unit for transportation applications. A second possible approach can be attributed to the work of [7], in which a probe observes the spacing between a probe and a leading vehicle. Based on Edie’s generalized definition, [7] estimate traffic density from vehicle traveled hours of probes (seconds) and the time-spacing information (sensing domain, spacing ×travel time (meter×second)). If a probe is able to sense multiple leading vehicles and two or more probes are connected, counting the number of vehicles between two probes can help estimate traffic density [7]. When we extend the functionality of a probe, as in the case with CAPs, it is easy to infer that a probe can capture all the data required for Edie’s definition to be applied.

Edie’s definition calculates traffic density from the relationship between vehicle hours traveled and time-space domain [8]. Traditional applications of Edie’s generalized definition limits their domain to have a canonical shape such as a rectangle. For instance, the time-space domain of a point-based density estimation appears with a rectangle shape (e.g., 15 sec×1 m). A snapshot-based estimation has an approximately zero-time interval. However, theoretically, it allows for the space-time domain to be any shape such as rectangular, circle, or a polygon. A moving probe shapes its time-space domain from the sensing distance and trajectory. CAPs also capture total vehicle traveled hours of detected vehicles, measuring how many vehicles are sensed in each time step.

Although this novel approach can capture the traffic density of a section, our simulation experiments show that the CAPs-based traffic density tends to over-estimate or under-estimate in certain situations. In the following section, we describe this limitation in detail and introduce our proposed LSTM neural network that is designed to overcome this limitation.

## 2. Preliminary

### 2.1. Specification of a Radar Sensor-Equipped Probe

One of our preliminary assumptions is that Connected and Autonomous Vehicles (CAVs) are equipped radar sensors to efficiently observe surrounding traffic conditions. Nam et al. [9] proposed a traffic density estimation method by utilizing a radar sensor equipped probe developed by Korea Institute of Civil Engineering and Building Technology (KICT). The probe vehicle has multiple sensors, including a GPS unit, high resolution radars working with 77-gigahertz microchips, and computing processors. Applications in the main processor manipulate incoming data from sensors and then compute local traffic density. The radar system of the probe vehicle is capable of observing multiple surrounding vehicles on a real-time basis and then tracing their trajectories, which are then used to calculate Vehicle Miles Traveled (VMT) and Vehicle Hours Traveled (VHT). VMT is defined as the product of traffic volume on a link and the length of the link. Similarly, VHT is defined as the total time traveled by all vehicles on a specific link during a specific period. Currently, the probe, called TRADOS (TRAffic Density Observation System), has an onboard real-time traffic monitoring tool, which can sense traffic conditions in its vicinity (e.g., local density).

**Figure 1:**
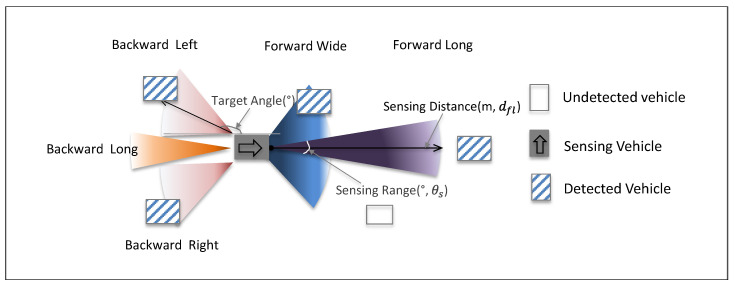
Sensing vehicle and its sensing range specifications.

Figure 1 shows a conceptual diagram of how the sensor-equipped vehicle detects adjacent vehicles. Radars are located at front and rear bumpers that detect vehicles in four regions surrounding the probe vehicle: front-end, rear-end center, left and right side. In the front, there are two radars: a forward long-range radar, and a mid-range radar, both of which are widely used for ACC applications. In our study, the long-range radar performs the role of detecting vehicles on the same lane, and the mid-range radar is adapted to detect vehicles on the left/right side lanes. The probe has three radars in the rear. Two side backward sensors designed for Blind Spot Detection (BSD) can detect vehicles driving in the left/right lanes. Finally, the backward long-range sensor is used to detect vehicles following the probe vehicle in the same lane.

Applications in the computing processors synchronize different sensor data on a time basis and store each detected vehicles’ relative position data, which is then converted into second-by-second vehicle trajectory data. By differentiating the trajectories with respect to time, we can infer the travel speed of detected vehicles. Similarly, the probe can also infer acceleration/deceleration of the detected vehicles, if required.

For this study, the following radar configuration is assumed as shown in Table 1.

### 2.2. Traffic Density Estimation and Its Limitations on the CAPs Application

Traffic density defined by Edie (1963) is as follows:

(1) $$\;\hat{K}\left(\left[x_{a},{\;x}_{b}\right]\times \left[t_{a},{\;t}_{b}\right]\right)=\frac{t\left(\left[x_{a},{\;x}_{b}\right]\times \left[t_{a},{\;t}_{b}\right]\right)}{\left[x_{a},{\;x}_{b}\right]\times \left[t_{a},{\;t}_{b}\right]}$$

where $t\left(\left[x_{a},{\;x}_{b}\right]\times \left[t_{a},{\;t}_{b}\right]\right)$ is the total vehicle hours travelled of all vehicles in the time-space domain ($\left[x_{a},{\;x}_{b}\right]\times \left[t_{a},{\;t}_{b}\right]$).

This definition can be reformulated for CAPs as Equation (2). We assume that a CAP observes adjacent vehicles at every time step j. J is the total travel time of a CAPs on a link. The space domain ($\left[x_{a},{\;x}_{b}\right]$) is simplified by the sensing performance of a CAP. The advantage of this method is that it does not calculate the local density every time step. Only a section density can be estimated by combining detected and current vehicles’ travel time until a sensor vehicle passes a domain.

(2) $$\;\hat{k}\left(\left[x_{a},{\;x}_{b}\right]\times \left[t_{a},{\;t}_{b}\right]\right)≒k\left(d_{s}\left[x_{a},{\;x}_{b}\right]\times \left[t_{a},{\;t}_{b}\right]\right)=\frac{\sum_{j=1}^{J}{\mathrm{VHT}}_{j}^{s}}{d_{S}\cdot J}=\frac{{\mathrm{VHT}}^{S}}{d_{S}\cdot J}$$

Although a CAP can capture traffic states during stationary traffic conditions such as non-congested and fully congested conditions, it is vulnerable during flow transition periods. Specifically, the performance deteriorates during the onset of congestion and queue-clearing conditions, in comparison with its performance in other states. 

**Figure 2:**
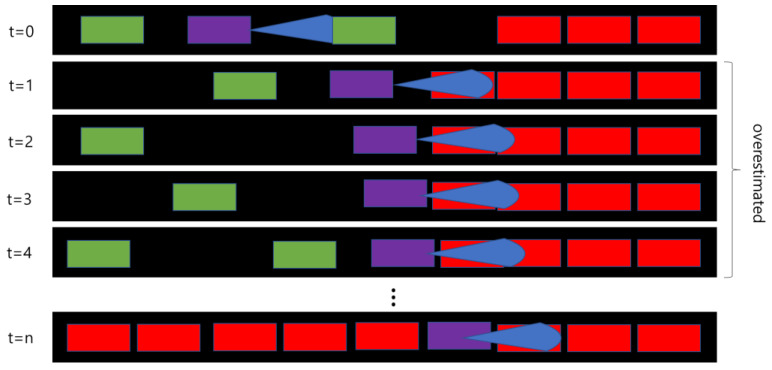
Overestimation pattern in time of onset of congestion.

Figure 2 shows a simplified illustration of overestimated traffic density during the onset of congestion, when a simple local density estimation algorithm is applied. The number of cars on the road section can be suitably thought of as a proxy for road density. The purple vehicles indicate CAPs, each having a sensing area shown in blue. Faster-moving and slower-moving vehicles are depicted in green and red, respectively. Depending on the sensor positions, the congestion build-up or clearing conditions can move through the sensing zone and cause a time-lag effect. A simple algorithm will naturally capture travel hours and travel distances of all vehicles in the sensing area of the CAPs to calculate densities, but during times of congestion there is a greater proportion of slower vehicles in the sensing area in each time step, which can cause oversampling. This oversampling of slower-moving vehicles from time step to time step can lead to overestimation of traffic density. To correct for this bias, the algorithm needs a certain time-step to time-step memory. Moreover, sensing areas from different CAPs tend to overlap in these conditions, which further add to the overestimation. The limitations of the algorithm outlined previously motivate us to incorporate innovative approaches to make our method more accurate. 

Estimating traffic density using multivariable data from CAPs is quite challenging since traffic conditions are dependent on complex temporal and spatial factors. Traffic density at the current time step is highly correlated with traffic conditions in the previous time steps, which motivates us to apply an estimation method that considers temporal dynamics of a series of input and outputs. The time dependency of traffic density varies by situation and entails stochastic characteristics. There are various domain specific algorithms for these conditions. Kalman filter [10], which is also known as linear quadratic estimation, is the representative method for real-time traffic state estimation [11,12,13,14,15,16].

Traffic density in transitional periods is too stochastic to estimate accurately using linear programing, resulting in overestimation during transitional periods. As previously indicated, traffic density tends to be overestimated since probe vehicles arriving at a congested part of a road section myopically classify the current link density as heavily congested even though the rest of the road section is not congested, which leads to estimation inaccuracies. Experimental results using simulation show that the traffic dynamics of congestion and queue clearing have recurrent patterns. The combination of highly recurrent patterns, nonlinearity, and a large amount of data to be analyzed leads us to propose Machine Learning (ML) algorithms as appropriate tools to identify these patterns. ML algorithms excel in extracting relationships between different variables in large datasets. In this research study, the data to be analyzed are a fusion of CAPs data and road geometry data. This fusion is not trivial, as we need to formulate innovative ways to combine these disparate sources of data. 

The capabilities of Deep Learning algorithms are increasingly being recognized in the fields of traffic estimation and prediction [17,18,19,20,21]. One common conclusion that has emerged in the field of traffic flow prediction is that deep learning methods are successfully able to identify nonlinearity of traffic flow according to time and time-lag characteristicsz and that deep learning algorithms can accurately predict traffic fundamentals. Recurrent Neural Networks [22] (RNN) have been utilized by [21,23,24,25] to predict traffic flows from prior traffic condition. RNN models, however, have a well-known limitation known as the “Vanishing Gradient Problem”. Due to this property, the effect of the prior steps’ traffic conditions tends to drastically weaken over time, thus deterring the implementation of time-lag properties of traffic dynamics; [17,18,21,26] point out this property and recommend Long-Short Term Memory Neural Networks [27], a scheme within the broad category of deep learning methods, as an effective tool to counteract this problem. They report that this LSTM approach could result in better estimation of traffic conditions and can better capture the time-lag properties of traffic dynamics.

Most recent LSTM applications in transportation research, however, focus on a traffic condition prediction where ground truth values at a certain location in prior timesteps are given and the model predicts the traffic condition of the next time step. In other words, the scope of contemporary research has been the prediction of traffic density at pre-defined fixed-point detectors. However, our scope of traffic density estimation is potentially network-level since our source of data are CAPs that travel throughout the network. With an assumption that we do not have a sectional level of ground truth traffic densities at every timestep, the proposed model estimates the ground truth traffic density by utilizing data from vehicles sensor and geo-spatial information. Thus, our traffic estimation process does not include previous step’s ground truth traffic densities as input variables. We propose a multivariate input variable-based LSTM neural network model that is explained in detail in the following section.

## 3. Deep Learning-Based Traffic Density Estimation

**Figure 3:**
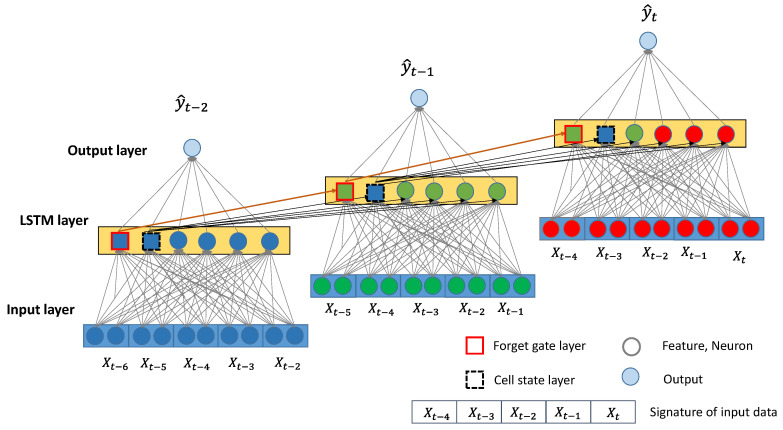
Design of LSTM for signature data from CAPs.

Figure 3 shows the conceptual framework of the proposed methodology. Hereinafter, we will call the proposed model as STREAM-LSTM (Simulation-based TRaffic density Estimation AlgorithM-Long-Short Term Memory). A traffic management center collects various pieces of information from CAPs passing a road section every 0.5 s and stores the data in a moving time horizon window vector of n time steps. In our study, we set the moving horizon to be five time steps. Traffic density is highly affected by prior traffic conditions. The time-horizon window has a pattern that is captured by CAPs during the moving horizon. We call this pattern a “Sensor Signature”. The LSTM network [18] recognizes the current signature (input layer) and previous time step’s layer pattern (LSTM layer). The advantage of employing LSTM networks in this context is that they do not suffer from the well-known “vanishing gradient” problem [17,18,19]. Simply stated, this means that they can consider not only quite recent prior conditions but also relatively longer prior conditions, as the phrase “Long Short-Term memory” implies. Our proposed framework takes the advantage of the memory layers that automatically determine the time lag between input and output. In traffic estimation domain, traffic conditions collected from CAPs might be locally biased since CAPs only capture adjacent traffic conditions. In other words, there exists a time lag between a CAP and actual traffic density when traffic is in a transitional period. Furthermore, some CAPs approaching congestion can be used as an indicator of increasing density. LSTM recognizes those time-lag conditions using temporally-varying Sensor Signatures.

This model can take multiple features as input variables from CAPs as shown in Figure 3. The model input vector at t is denoted as $x_{t}=\left(x_{1,t},x_{2,t},x_{3,t},\cdots ,x_{\tau ,t}\right).$ A neuron ($i_{t}$) in the input layer at time t (Equation (3)) consists of an input ($x_{i,\;t}$), a hidden vector of previous time step ($h_{t-1}$), weights for the two vectors ($W_{i},\;U_{i})$, and bias $b_{i}$. To reflect time-relevant characteristics in the data, this model uses a “forget gate layer” (Equation (4)) and a “cell state layer” (Equation (5)) to store temporal information, which is the output of neuron states in the previous time step. Forget layer f is called a transfer function that is determined to be either forgotten or alive from the previous states by cell state layer. For example, the forget layer in our method determines the time period of considering time lags between sensor input and traffic density. If the sensor data of past time periods does not affect the density of the current time step, the cell state layer decides to not use the forget layer. The function can take any form such as linear, sigmoid, tanh, or ReLU. From each neuron state, density is estimated by Equation (6)

(3) $$i_{t}=\sigma_{g}\left(W_{i}x{\;}_{i,t}+U_{i}h_{t-1}+b_{i}\right)$$

(4) $$f_{t}={\;\sigma }_{g}\left(W_{f}x_{t}+U_{f}h_{t-1}+b_{f}\right)$$

(5) $$c_{t}=f_{t}{}^{\circ}c_{t-1}+i_{i}{}^{\circ}{\;\sigma }_{c}\left(W_{C}x_{t}+U_{c}h_{t-1}+b_{c}\right)$$

(6) $$\hat{y}\left(t\right)=f(\sum_{i}^{I}w_{i}n_{i}\left(t\right)+b_{o})$$

where

$x_{t}:$ Input vector at t
$h_{t-1}$: a hidden vector of previous time step
$i_{t}\;$: Input layer at t
$c_{t}:$ Cell state layer at t
$f_{t}\;$: Forget gate layer at t
${\hat{y}}_{t}:$ Estimated output at t
$\sigma_{h},{\;\sigma }_{y}$: Activation function
$W_{h},\;U_{h}$: Weights of a layer h (it plays a role in connecting perceptrons among layers)
$b_{h},\;b_{c},\;b_{o}$: Bias vector

**Figure 4:**
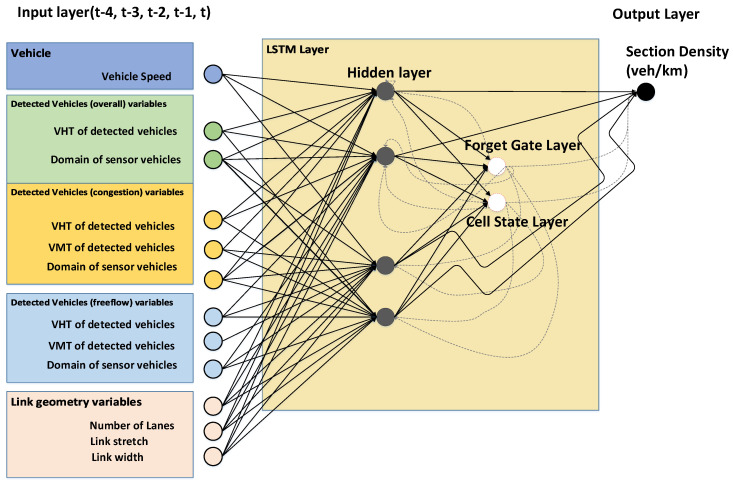
Input-output variables and LSTM network design.

**Figure 5:**
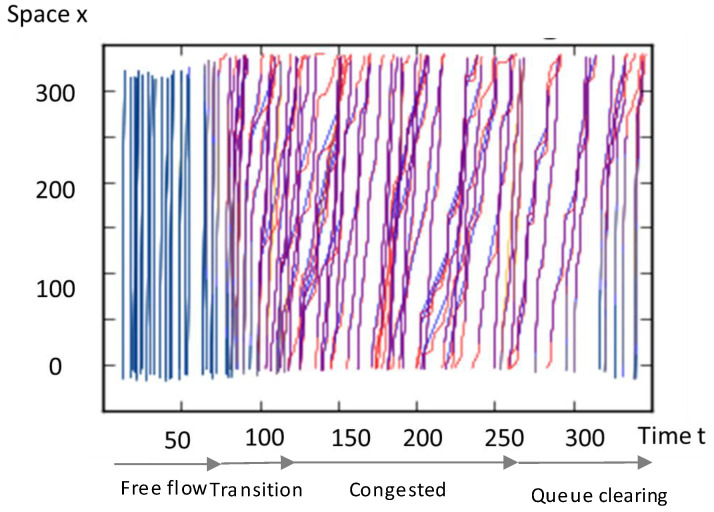
Vehicle trajectory variation according to the congestion level.

After multiple experiments, we select various input variables that are known to have an effect on traffic density, as shown in Figure 4. First, we consider the travel speed of a sensor vehicle. We refer to the well-known Edie’s definition that traffic density is a function of vehicle travel time over a time-space domain. The traffic pattern in non-congested conditions is significantly different from that in congested conditions, as can be seen in Figure 5. With this insight, we categorize the traffic condition into two regimes and calculate the variables (VHT, VMT, Sensor time-space domain) in each traffic regime. We set the congestion criterion of the expressway in this study to 80 km/hour.

## 4. Microscopic Simulation Based Evaluation

### 4.1. Simulation Environment

**Figure 6:**
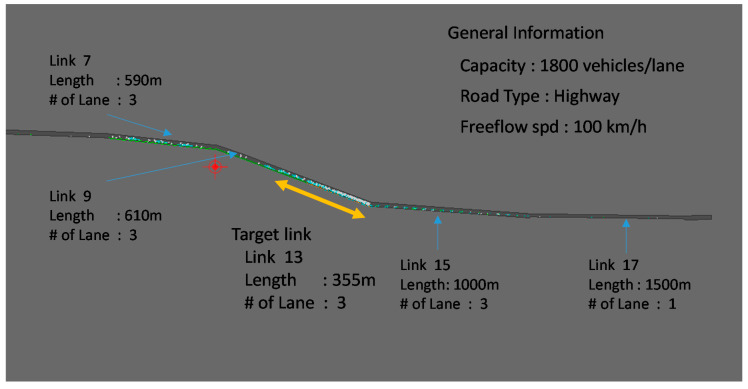
The overview of the hypothetical network.

This research evaluates the proposed method by using a microscopic simulation program (PARAMICS). The testbed for our evaluation is a simple traffic network shown in Figure 6, hereafter called the hypothetical network. This network contains one single stretch of freeway, with three lanes, and one origin-destination pair. The entire simulation period is set to be 1.5 h, out of which the first 0.25 h and the last 0.25 h are discarded due to the peculiarities of the simulation software where we have previously observed erratic vehicle behavior around the boundary conditions (beginning and end of the simulation). The period of evaluation is set to be the middle 1 h.

The demand on this O-D pair is set to be 5000 vehicles for the simulation period. For our study, congestion is artificially induced in the network by dropping the number of lanes abruptly from three lanes on link 13 to two lanes on its immediate downstream link 11. The network was simulated for different market penetration of sensor vehicles, and for various sensor-vehicle characteristics, such as sensing distance, target angle, etc. Table 2 and Table 3 indicate the sensor configuration in the simulation and evaluation configuration, respectively.

### 4.2. Results

The complete dataset is segregated into two parts: training data and test data. These datasets contain information collected by the CAPs and have both static (link geometry) and dynamic (vehicle sensed, link flow, etc.) information. The training dataset is used by the model to build relationships between link density and data from CAPs. The trained model is then deployed on the test data to evaluate its accuracy. This procedure is repeated for different market penetration ratios. 

**Figure 7:**
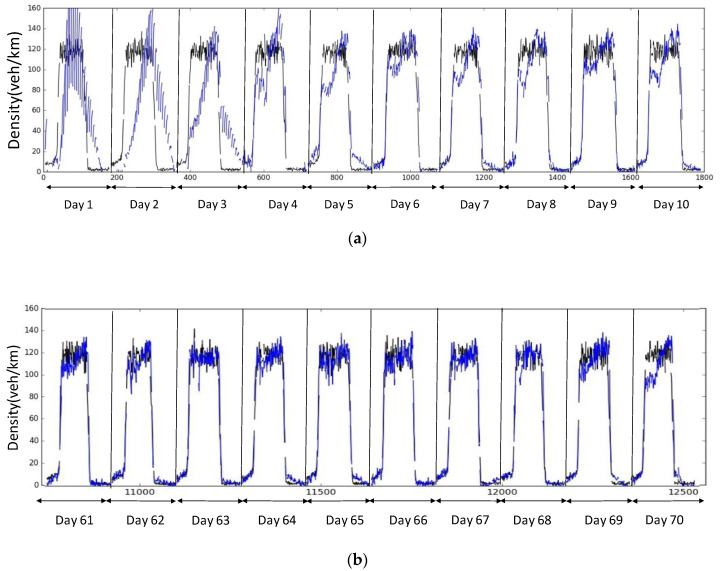

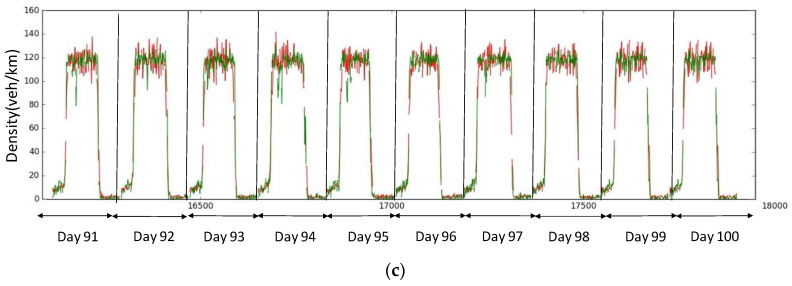
Training Process and the evaluation with the test data set. (**a**) Training Process for day 1 to 10 (link 13); (**b**) Training process for day 61 to 70 (link 13); (**c**) Test results for day 91 to 100 (link 13).

Figure 7 shows how the model is trained on data obtained from a specific link (link# 13) on a simple hypothetical network, when the sensor vehicles have a market share of 25%. The *x*-axis and *y*-axis represent learning time and estimated densities, respectively. Note that the data here are simulated, which means data from different training runs could conceivably be thought of as traffic data from different days. Real density is shown in the black line, while the estimated density is shown in blue in Figure 7a,b), and green in Figure 7c. Specifically, Figure 7a shows the training over the first 10 days. We can see that in the first few runs, the algorithm is not very accurate in estimating density, but that is to be expected, as it needs more data to correct itself. The degree of accuracy is significantly improved from repeated learning (Figure 7b), as is evident from noting the difference between Run# 1 and, say, Run# 6 onwards.

**Figure 8:**
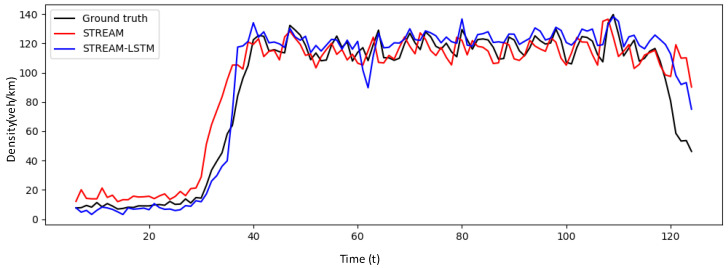
Overall comparisons between the proposed STREAM-LSTM and STREAM.

Figure 8 indicates the estimated densities averaged over the test runs of our proposed method in comparison with STREAM, which uses Edie’s generalized definition detailed in Equation (1). The performance of the STREAM-LSTM is compared with that of a density estimation method of STREAM. The proposed methodology shows an improvement over Edie’s method (STREAM). The simulation period of 1 hour is characterized by various flow regimes and it would be instructive to observe the performance of our algorithm in different traffic conditions.

**Figure 9:**
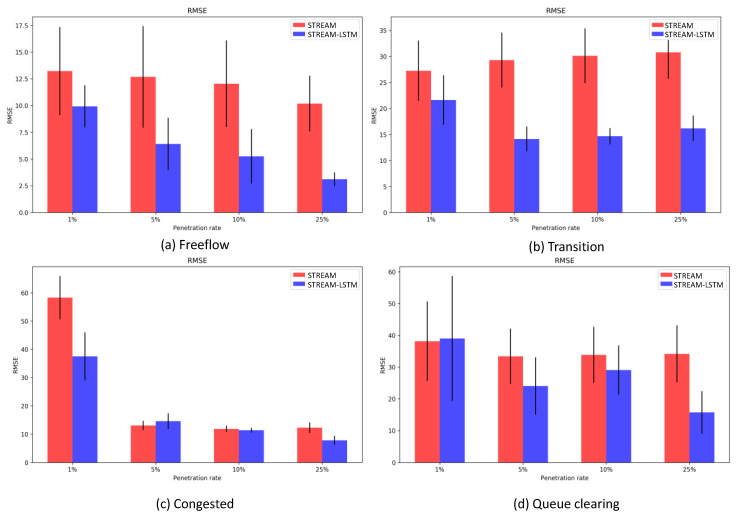
RMSE comparison in different flow regimes.

The simulation period can be roughly divided into four flow regimes: free-flow conditions, transition from free flow to congested conditions, congested condition, and queue-clearing condition at the very end (Figure 8). As expected, STREAM (red line) tends to estimate traffic density poorly at the onset of congestion and during queue-clearing conditions. Our model accurately estimates traffic density in Free-flow, Transition, and Congested conditions (Figure 9). Although overestimation still remains a problem in the Queue clearing condition, the STREAM-LSTM method converges to the actual density faster than STREAM.

Our evaluation results confirm that the proposed method performs better than previous methods. This is because our proposed method fully utilizes the signature of multiple forms of information gathered from multiple CAPs. Additionally, LSTM Neural Networks can efficiently memorize the relationship between the signature and time-lag characteristics of traffic densities.

By means of comparison, we also calculated the estimated densities on link 13 over the same test dataset using Edie’s definition. The numerical values for Root Mean Squared Error and Relative Error using both methods at different penetration rates are shown in Table 4. The performance gets better as the penetration rate increases, with an almost 45% improvement in RMSE and 66% improvement in Relate Error in the 25% market penetration scenario.

## 5. Conclusions

In this research study, we proposed an LSTM approach, which is a non-parametric method to estimate traffic density. This research was partially motivated by CAPs technology and current research literature on probe-based traffic density estimation methods. Our simulation analysis shows that a mathematical algorithm (STREAM) tends to overestimate density in certain traffic conditions. This study proposes a method to estimate the traffic density for a single link using a STREAM-LSTM model. The performance of the STREAM-LSTM method is compared with that of a density estimation method of STREAM. 

Our model accurately estimates traffic density in Free-flow, Transition, and Congested conditions. Although overestimation still remains a problem in the queue-clearing condition, the STREAM-LSTM method converges to the actual density faster than STREAM. This is because our proposed method fully utilizes the signature of multiple forms of information gathered from CAPs. The other reason is that LSTM Neural Network can efficiently memorize the relationship between the signature and time-lag characteristics of traffic densities. Our research focuses on the accuracy of traffic density estimation in the domain of CAPs and evaluates the proposed method by multiple simulation runs. Statistically reliable information is also an important aspect in traffic management and advanced traffic information systems. There are reasonable methods to capture reliability such as Monte-Carlo simulations, which is a direction for our future research. Factors affecting traffic conditions such as geometry and heterogeneity of vehicles have not been considered in this study, but are under consideration in our current and future research.

It is evident that the traffic characteristics of traffic flow on one link have an impact on those nearby links. Therefore, it stands to reason that if we formulate these relationships between links and present those as additional input data to our LTSM model, its accuracy can be expected to improve. 

In addition, because traffic density has a close relationship with other fundamental traffic flow variables such as flow and speed, a high degree in the estimation of density brings us one step closer to more accurately estimating these other performance indicators as well. Our results show that the proposed model outperforms existing methods. However, there is still variation in its performance under different traffic conditions. There are some traffic conditions, such as queue clearing, where the proposed model’s performance is better than mathematical models, but not by a significant amount. Our model can also be extended, without significant computational overhead, to a multiple link scenario. However, it may be necessary to design an evolution type LSTM using input data of three-dimensional tensor-type data. Lastly, the proposed method can be used in the real-time lane-wise queue-length estimation and prediction in urban road networks, which should be one of the crucial messages for the safety and efficiency of the connected and automated vehicles in the near future.

## Figures and Tables

**Table 1:** Radar configuration of Connected and Autonomous Probes.

Sensor Code	Name	Target Angle (°)	Sensing Range (°)	Sensing Distance (m)
1	Forward Long	0	10	30
2	Forward Wide	0	60	10
3	Backward Left	160	40	10
4	Backward Right	200	40	10
5	Backward Long	180	20	20

**Table 2:** Sensor configuration.

Sensor Code	Name	Target Angle	Range	Distance
**1**	Front Long	0	10	30
**2**	Front Short	0	60	10
**3**	Rear Left	160	40	10
**4**	Rear Right	200	40	10
**5**	Rear Center	180	20	20

**Table 3:** Configuration for performance evaluations.

Type	Name	Configuration
**Simulation**	Simulation time	1.5 h
	Warm up time	First 0.25 h
	Analysis time	1 h
	Updating time step of simulation	0.1 s
	Demand profile	5000 vehicles/1.5 h
	Vehicle compositions	-Sensor vehicle (1% to 10%) -Regular vehicle
	Generated samples	100 days of morning peaks
**Density Estimation**	Updating time step	30 s
	Congestion criteria	80 km/hour
	Size of the moving horizon	5-time step (2 min 30 sec)
	Dataset composition	Training: 70 days Test: 30 days

**Table 4:** Evaluation results for the proposed method (link 13).

Penetration Rate	RMSE			Relative Error		
	STREAM-LSTM	STREAM	Improve (%)	STREAM-LSTM	STREAM	Improve (%)
1%	32.69	49.15	33.50	0.36	0.50	27.80
5%	14.08	16.29	13.54	0.18	0.30	40.14
10%	12.12	15.69	22.74	0.15	0.30	49.68
25%	8.88	15.97	44.36	0.11	0.34	66.11
